# Tofacitinib induces G1 cell-cycle arrest and inhibits tumor growth in Epstein-Barr virus-associated T and natural killer cell lymphoma cells

**DOI:** 10.18632/oncotarget.12529

**Published:** 2016-10-08

**Authors:** Shotaro Ando, Jun-ichi Kawada, Takahiro Watanabe, Michio Suzuki, Yoshitaka Sato, Yuka Torii, Masato Asai, Fumi Goshima, Takayuki Murata, Norio Shimizu, Yoshinori Ito, Hiroshi Kimura

**Affiliations:** ^1^ Departments of Pediatrics, Nagoya University Graduate School of Medicine, Nagoya 466-8550, Japan; ^2^ Departments of Virology, Nagoya University Graduate School of Medicine, Nagoya 466-8550, Japan; ^3^ Departments of Pathology, Nagoya University Graduate School of Medicine, Showa-ku, Nagoya 466-8550, Japan; ^4^ Center of Stem Cell and Regenerative Medicine, Tokyo Medical and Dental University, Chiyoda-ku, Tokyo 101-0062, Japan

**Keywords:** tofacitinib, EBV, lymphoma, cell-cycle arrest

## Abstract

Epstein-Barr virus (EBV) infects not only B cells, but also T cells and natural killer (NK) cells, and is associated with T or NK cell lymphoma. These lymphoid malignancies are refractory to conventional chemotherapy. We examined the activation of the JAK3/STAT5 pathway in EBV-positive and -negative B, T and NK cell lines and in cell samples from patients with EBV-associated T cell lymphoma. We then evaluated the antitumor effects of the selective JAK3 inhibitor, tofacitinib, against these cell lines *in vitro* and in a murine xenograft model. We found that all EBV-positive T and NK cell lines and patient samples tested displayed activation of the JAK3/STAT5 pathway. Treatment of these cell lines with tofacitinib reduced the levels of phospho-STAT5, suppressed proliferation, induced G1 cell-cycle arrest and decreased EBV LMP1 and EBNA1 expression. An EBV-negative NK cell line was also sensitive to tofacitinib, whereas an EBV-infected NK cell line was more sensitive to tofacitinib than its parental line. Tofacitinib significantly inhibited the growth of established tumors in NOG mice. These findings suggest that tofacitinib may represent a useful therapeutic agent for patients with EBV-associated T and NK cell lymphoma.

## INTRODUCTION

The Epstein-Barr virus (EBV) is a ubiquitous virus that has infected up to 95% of the adult population worldwide. After primary infection, EBV establishes a latent infection for the entire lifetime of the host. Although EBV predominantly infects B cells, it also infects T or NK cells, especially in association with lymphoid malignancies including NK/T-cell lymphoma, hydroa vacciniforme-like lymphoma, and chronic active EBV disease (CAEBV) [1–4]. As some types of EBV-associated lymphoma are often refractory and resistant to conventional chemotherapies, various novel treatments have been attempted. Although some progress has been achieved with treatments such as rituximab (a humanized monoclonal antibody against CD20) and EBV-specific cytotoxic T lymphocyte infusion, the effects of those new therapies are still restricted. Therefore, novel approaches involving targeted molecular therapies are desirable, especially for T or NK cell malignancies [5–9].

The Janus kinase (JAK)-signal transducers and activators of transcription (STAT) pathway is one of the critical intracellular cascades in transduction of extracellular signals from cytokines and growth factors. Various physiological functions such as cellular growth, differentiation and immune regulation are controlled by the JAK/STAT pathway [10, 11]. JAKs, comprising JAK1, JAK2, JAK3 and tyrosine kinase 2 (TYK2), are cytoplasmic non-receptor tyrosine kinases. They associate with specific cytokine receptor chains and transduce signals by phosphorylating tyrosine residues on STAT transcription factors. Dysregulation of the JAK/STAT pathway has been described in various human diseases such as in malignant, immunological, and inflammatory diseases [12, 13].

The JAK family member JAK3 is specifically related to T cell development and proliferation. Recent data have also indicated that JAK3 activating mutations are found in human hematological malignancies including NK/T-cell lymphoma [14–16]. Unlike other JAK family members, JAK3 is predominantly expressed in hematopoietic tissues and is associated with the common gamma-chain. This restricted expression and function make JAK3 an attractive therapeutic target [11, 17].

Tofacitinib, a pyrrolo-pyrimidine JAK3-selective inhibitor, was originally developed as an orally active immunosuppressant, and has already received FDA approval for the treatment of rheumatoid arthritis [18, 19]. Concurrently the potential use of tofacitinib as an anticancer agent has recently begun to garner attention, and several studies have demonstrated antitumor effects of tofacitinib against various types of malignancies including EBV-positive NK cell lymphoma cells [14, 15].

In this study, we investigated the activity of the JAK3/STAT5 pathway in EBV-associated B, T and NK cell lines as well as in lymphocytes from patients with EBV-associated T cell lymphoma, and evaluated the antitumor effects of tofacitinib on those cell lines in culture and in a murine xenograft model.

## RESULTS

### The JAK3/STAT5 pathway is activated in EBV-positive T and NK cell lines and tofacitinib inhibits its activation

**Figure 1:**
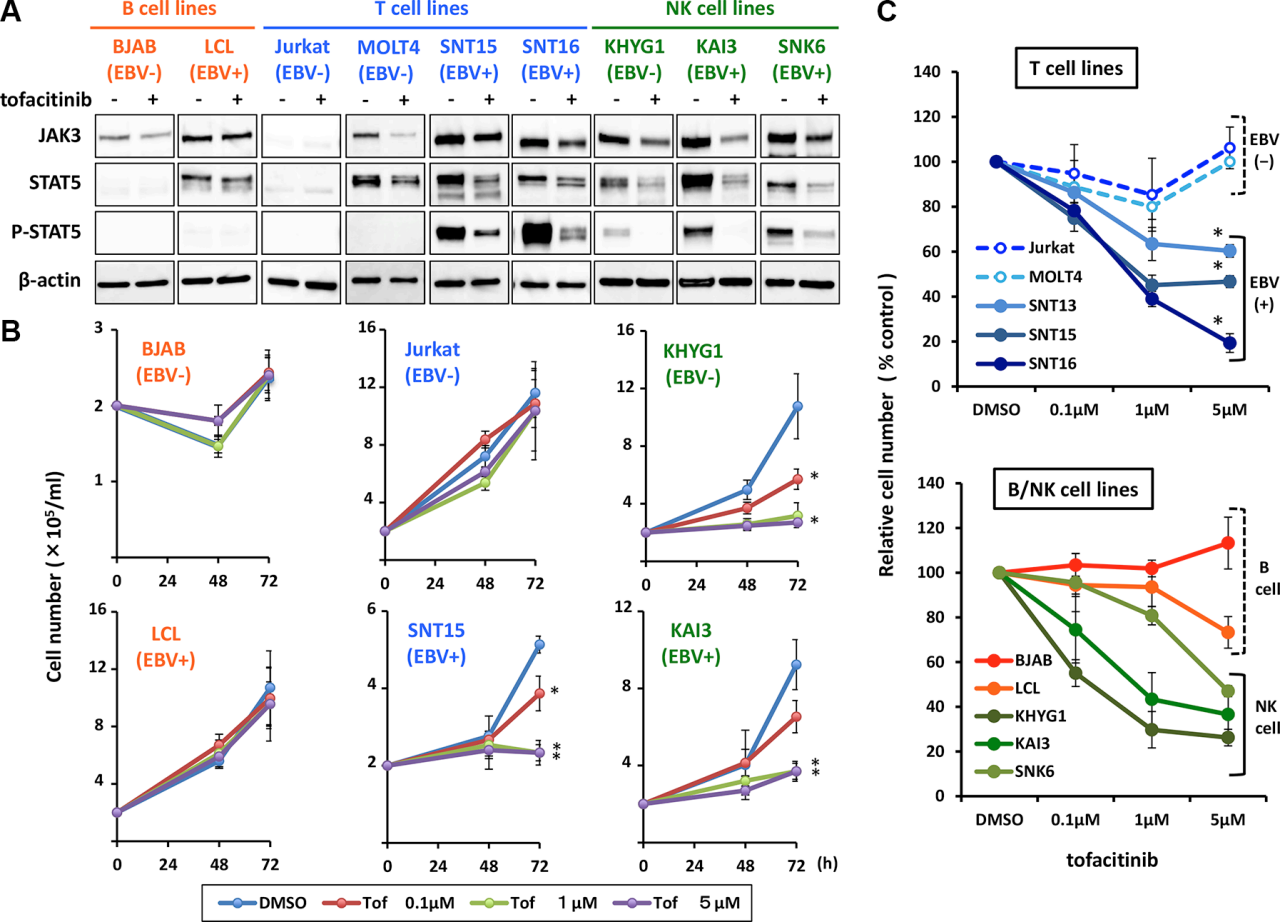
Effects of tofacitinib on JAK3/STAT5 pathway components and growth in B, T and NK cell lines (**A**) The following cell lines: EBV-negative B cell line (BJAB), EBV-transformed lymphoblastoid cell line (LCL), EBV-negative T cell lines (Jurkat and MOLT4), EBV-positive T cell lines (SNT15 and SNT16), EBV-negative NK cell line (KHYG1), and EBV-positive NK cell lines (KAI3 and SNK6), were treated without (−) or with (+) 1 μM tofacitinib for 24 h and cell lysates were then immunoblotted for the indicated proteins. (**B**) BJAB, LCL, Jurkat, SNT15, KHYG1 and KAI3 cells were treated with the indicated concentrations of tofacitinib, and viable cells were counted at the indicated times using the trypan blue exclusion test. Values are means ± SE of the results from triplicate experiments. *P < 0.05 as compared with DMSO-treated cells. (**C**) B, T and NK cell lines were treated with the indicated concentrations of tofacitinib for 72 h. Cell number is shown as the ratio of the cell number in the different treatment groups to DMSO-treated cells. Values are means ± SE of the results from triplicate experiments. *P < 0.05 as compared with Jurkat or MOLT4.

To determine the presence and activation status of the JAK3/STAT5 pathway in EBV negative or positive B, T and NK cell lines, we examined the expression of JAK3, and the expression and phosphorylation status of STAT5, in an EBV-negative B cell line (BJAB), an EBV-transformed lymphoblastoid cell line (LCL), EBV-negative T cell lines (Jurkat and MOLT4), EBV-positive T cell lines (SNT13,15 and 16), an EBV-negative NK cell line (KHYG1), and EBV-positive NK cell lines (KAI3 and SNK6). The characteristics of each cell line are summarized in Table 1. As shown in Figure 1A, phospho-STAT5 was not detected in the B cell lines, regardless of EBV status, although JAK3 expression was detected. In contrast, phospho-STAT5 was detected in the EBV-positive T and NK cell lines, suggesting activation of the JAK3/STAT5 pathway. Weak phosphorylation of STAT5 was detected in the EBV-negative NK cell line, KHYG1. Phospho-STAT5 was not detected in EBV-negative T cell lines (Jurkat and MOLT4) (Figure 1A). Treatment of the cell lines with tofacitinib inhibited the observed phosphorylation of STAT5 and decreased the expression levels of JAK3 and STAT5.

**Table 1:** Characteristics of the cell lines

Cell type	Cell line	EBV	Cell origin
B cell lines	BJAB	–	Burkitt lymphoma
	LCL	+	Primary B cells transformed with EBV
T cell lines	Jurkat	–	Acute T lymphoblastic leukemia
	MOLT4	–	Acute T lymphoblastic leukemia
	SNT13	+	CAEBV
	SNT15	+	CAEBV
	SNT16	+	CAEBV
NK cell lines	KHYG1	–	Aggressive NK cell leukemia
	KAI3	+	CAEBV
	SNK6	+	NK/T cell lymphoma
	NKL	–	Large granular lymphocyte leukemia
	TL1	+	NKL cell line

LCL; lymphoblastoid cell line, CAEBV chronic active EBV disease.

### Inhibition of the JAK3/STAT5 pathway by tofacitinib suppresses the growth of EBV-positive T cell lines and EBV-positive and negative NK cell lines

To determine whether the growth of B, T and NK cell lines was sensitive to tofacitinib, the cells were exposed to 0.1 to 5 μM of tofacitinib, and cell counts were determined after 48 and 72 h. Neither fresh medium nor additional drugs were added during the observation period. As shown in Figure 1B and 1C, EBV-positive T cell lines were significantly more sensitive to tofacitinib than EBV-negative T cell lines. At 5 μM of tofacitinib, the cell number of each EBV-positive T cell line was significantly lower relative to that of Jurkat or MOLT4 (Figure 1C). As for B cell lines, tofacitinib did not significantly inhibit their proliferation, regardless of EBV status (Figure 1B and 1C). Regarding NK cell lines, although tofacitinib suppressed cell growth, no differences were observed between EBV-positive and –negative cell lines (Figure 1B and 1C). Overall, when compared with untreated cells, inhibition of cell proliferation by tofacitinib was significant at a concentration greater than 1 μM.

**Figure 2:**
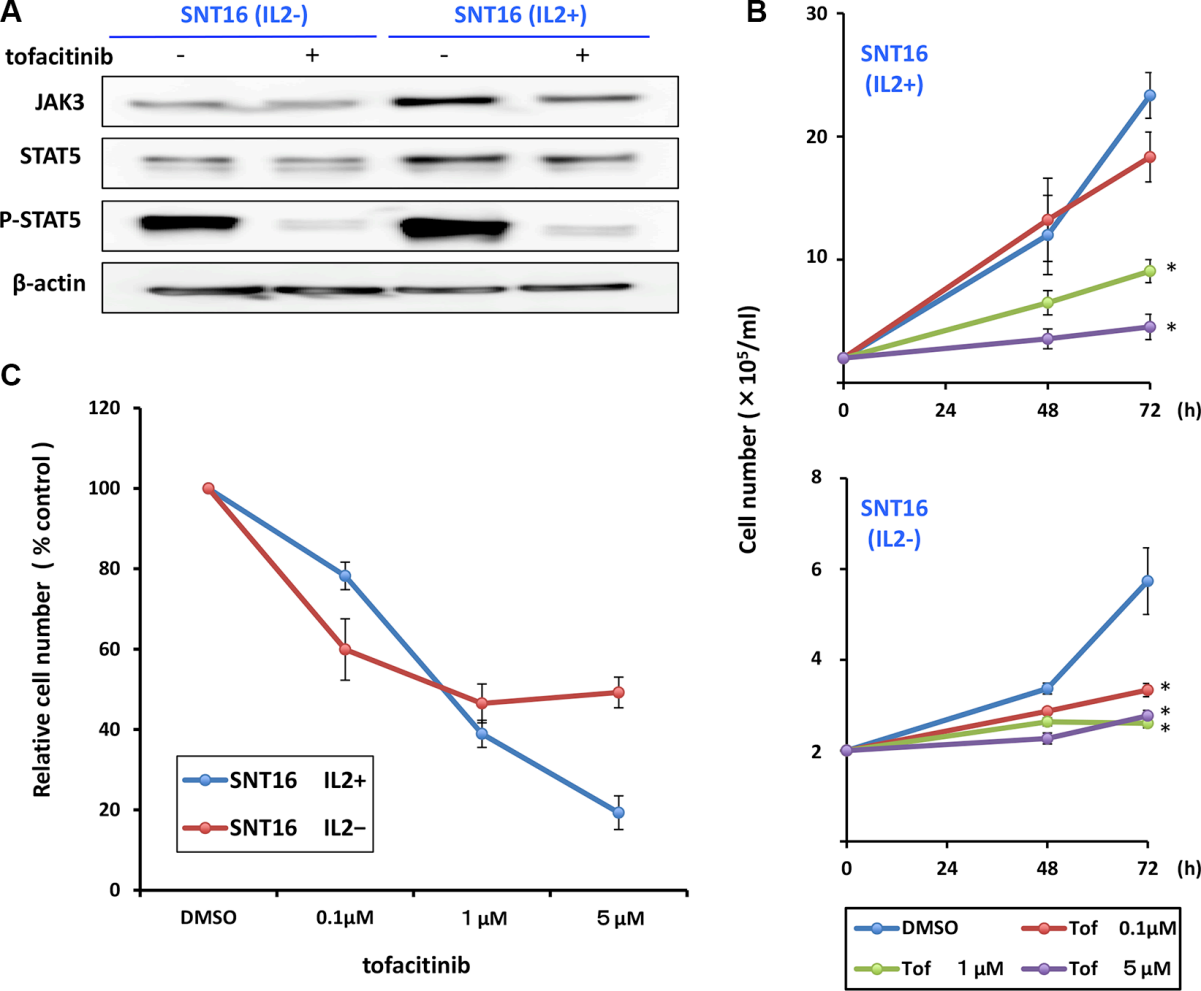
Effects of tofacitinib on JAK3/STAT5 pathway components and growth of IL2-dependent and -independent SNT16 cell lines (**A**) IL2-dependent and -independent SNT16 cells were treated with 1 μM tofacitinib for 24 h and cell lysates were immunoblotted for the indicated proteins. (**B**) SNT16 cells were treated with the indicated concentrations of tofacitinib, and viable cells were counted using the trypan blue exclusion test. Values are means ± SE of the results from triplicate experiments. *P < 0.05 as compared with DMSO-treated cells. (**C**) SNT16 cells were treated with the indicated concentrations of tofacitinib for 72 h. Cell number is shown as the ratio of the cell number in the different treatment groups to DMSO-treated cells.

Additionally, to examine the effect of IL-2 on JAK3/STAT5 pathway activation, we compared the expression of phospho-STAT5 in IL-2-independent and -dependent SNT16 cell lines. As shown in Figure 2A, phospho-STAT5 was detected in both cell lines and treatment with tofacitinib decreased the level of phospho-STAT5 in both cell lines. Moreover, tofacitinib inhibited the growth of the IL-2-independent SNT 16 cell line to the same extent as the IL-2-dependent cell line, although cell proliferation of the IL2-independent SNT16 cells was less than that of the IL-2 dependent SNT16 cells (Figure 2B and 2C).

### The presence of EBV in an NK cell line increases its susceptibility to tofacitinib

**Figure 3:**
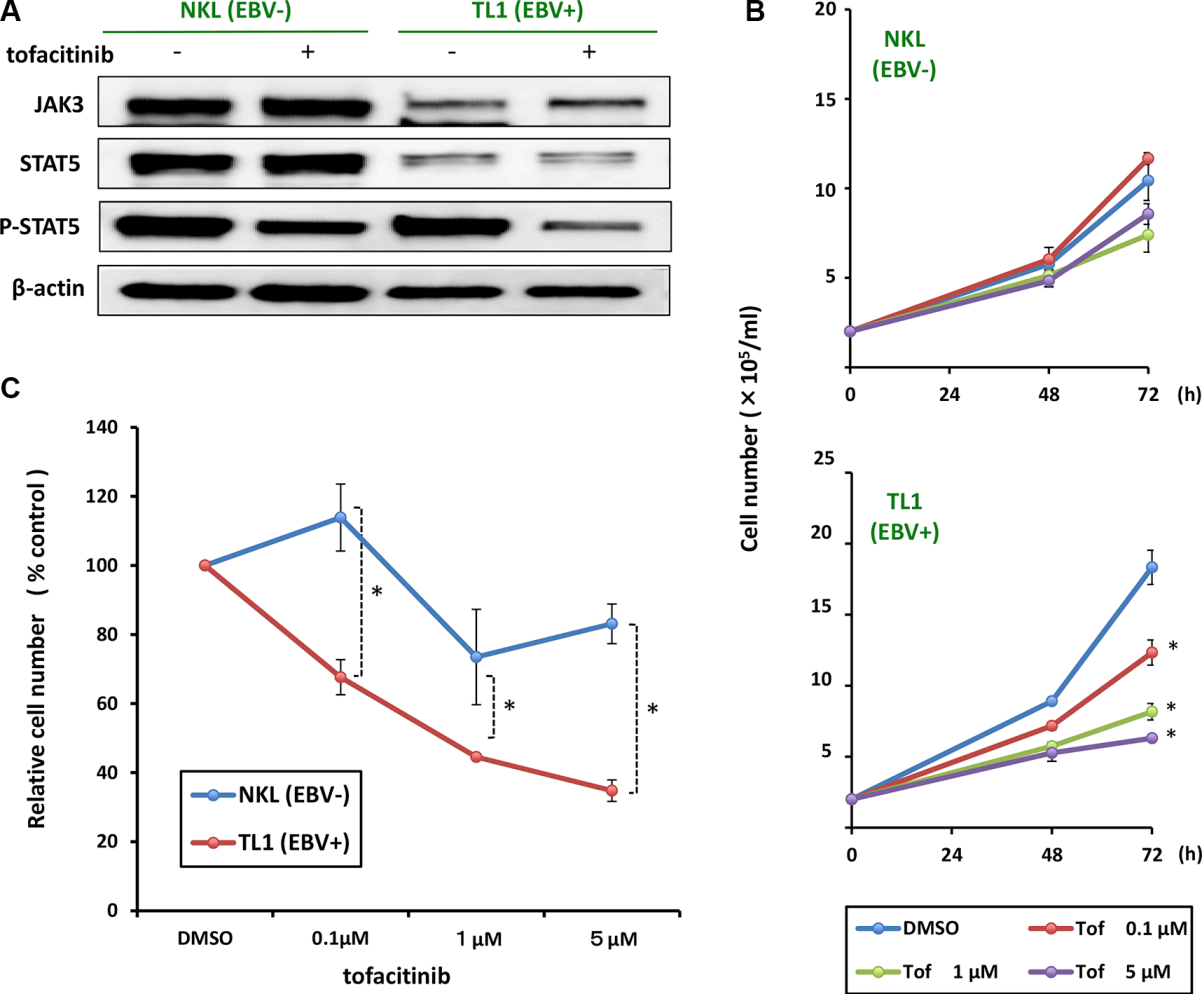
Effects of tofacitinib on JAK3/STAT5 pathway components and growth in NKL/TL1 cell lines (**A**) NKL and TL1 cell lines were treated with 1 μM tofacitinib for 24 h and cell lysates were immunoblotted for the indicated proteins. (**B**) The EBV-positive cell line (TL1) and its parental EBV negative cell line (NKL) were treated with the indicated concentrations of tofacitinib, and viable cells were counted using the trypan blue exclusion test. Values are means ± SE of the results from triplicate experiments. *P < 0.05 as compared with DMSO-treated cells. (**C**) TL1 and NKL cells were treated with the indicated concentrations of tofacitinib for 72 h. Cell number is shown as the ratio of the cell number in the different treatment groups to DMSO-treated cells. Values are means ± SE of the results from triplicate experiments. *P < 0.05 as compared with DMSO-treated cells.

To directly compare the effects of tofacitinib on EBV-positive and -negative cells, we administered tofacitinib to the EBV-positive NK cell line, TL1, and to the EBV-negative parental cell line, NKL. As shown Figure 3A, we found that the reduction in the expression of phospho-STAT5 by treatment with tofacitinib was greater in TL1 cells than in NKL cells, although there were no differences between TL1 and NKL cells with regard to change in JAK3 or STAT5 expression. Furthermore, TL1 cells were more sensitive to tofacitinib induced inhibition of proliferation than NKL cells (Figure 3B and 3C).

### Tofacitinib induces apoptosis in certain T and NK cell lines

**Figure 4:**
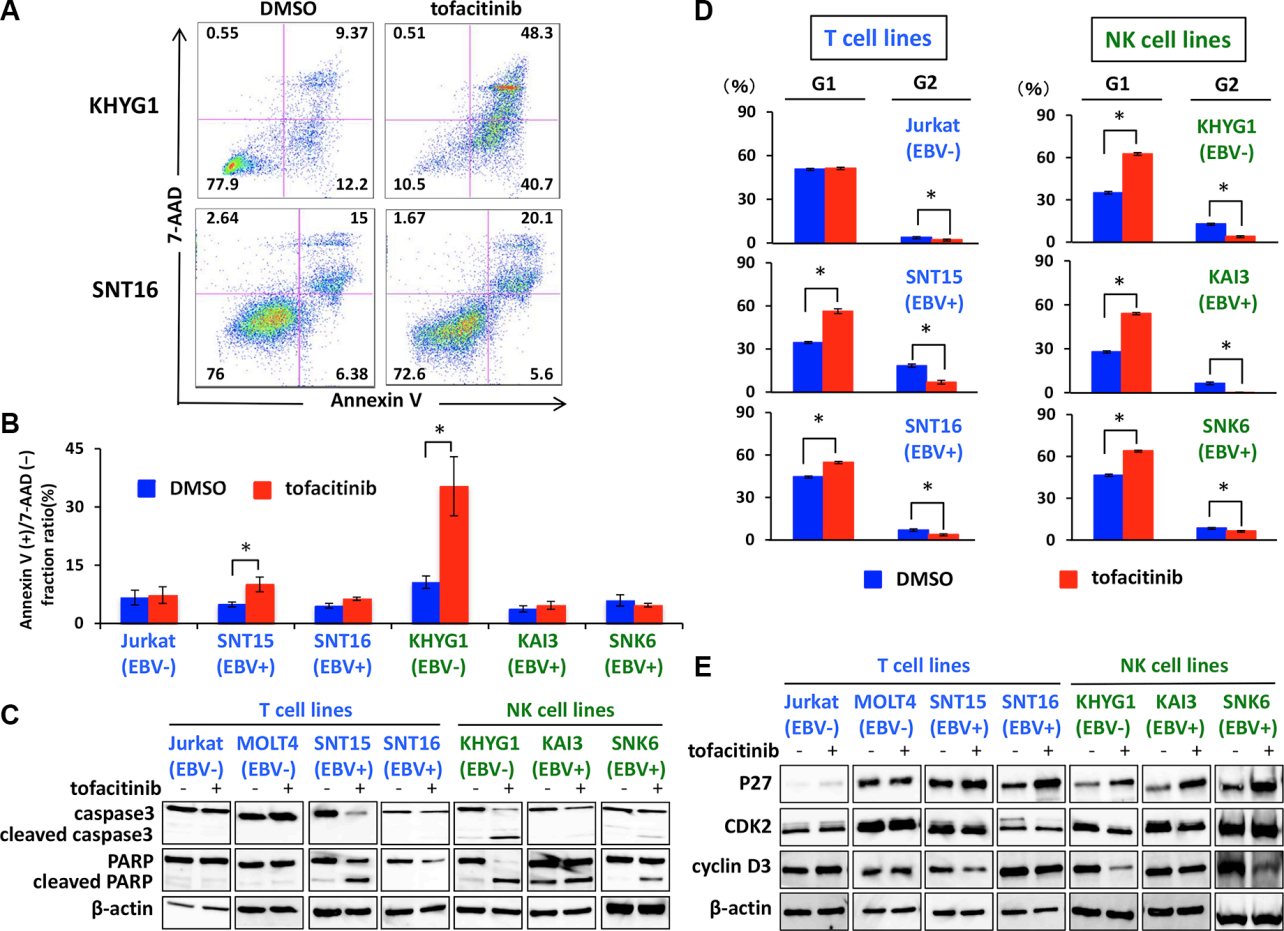
Effects of tofacitinib on apoptosis and cell-cycle arrest in T and NK cell lines (**A**, **B**) T and NK cell lines were treated with DMSO or 5 μM tofacitinib for 48 h, and apoptosis was evaluated by Annexin V-7AAD staining using flow cytometry. Values are means ± SE of the results from duplicate or triplicate experiments. *P < 0.05 as compared with DMSO-treated cells. (**C**) T and NK cell lines were treated with 5 μM tofacitinib for 48 h, and cell lysates were immunoblotted for caspase-3 and PARP. (**D**) T and NK cell lines were treated with DMSO or 5 μM tofacitinib for 24 h, following which they were fixed and stained with propidium iodide. Cell cycle profiles were assessed using flow cytometry. Values are means ± SE of the results from triplicate experiments. *P < 0.05 as compared with DMSO-treated cells. (**E**) T and NK cell lines were treated with 5 μM tofacitinib for 24 h and cell lysates were immunoblotted for the indicated proteins.

To evaluate whether tofacitinib induces apoptosis, apoptosis of various tofacitinib-treated cells was analyzed by flow cytometry after Annexin V staining. Tofacitinib treatment of KHYG1, an EBV-negative NK cell line, induced a significant increase in apoptotic cells, as shown by an increase in the Annexin V-positive and 7-AAD-negative fraction, when compared with dimethyl sulfoxide (DMSO)-treated cells (Figure 4A and 4B). A significant increase in apoptotic cells was also observed following tofacitinib treatment of SNT 15 cells, an EBV positive T cell line, although the frequency of apoptotic cells was much lower than that of KHYG1 apoptotic cells (Figure 4B). On the other hand, no significant increase in the number of apoptotic cells was observed in the other cell lines tested (Figure 4A and 4B). In addition, these cell lines were treated with 5 μM of tofacitinib for 48 h, and apoptosis was assessed by analysis of the cleavage of caspase-3 and PARP by immunoblotting. Consistent with the results obtained using flow cytometry, increased levels of cleaved caspase-3 together with decreased levels of full-length caspase-3 were observed only in KHYG1 cells. Although increased levels of cleaved PARP were observed in SNT15 and SNK6 cells, decreased levels of full-length PARP were modest, and faint or no bands of cleaved caspase 3 were observed in these cell lines (Figure 4C). The combined results suggest that tofacitinib prominently induces apoptosis in the EBV-negative NK cell line, whereas it induces modest or no apoptosis in the EBV-positive T and NK cell lines.

### Tofacitinib induces G1 cell-cycle arrest in T-and NK-cell lines

We next investigated whether growth inhibition of T and NK cells by tofacitinib was a result of cell-cycle arrest. T and NK cells were treated with 5 μM of tofacitinib for 24 h, were stained with propidium iodide, and were then analyzed using flow cytometry. Both an increase in cells in the G1 phase and a decrease in cells in the G2 phase were observed after treatment of SNT15, SNT16, KHYG1, SNK6, and KAI3 cells with tofacitinib (Figure 4D). Consistent with G1 cell cycle arrest, increased levels of the cyclin dependent kinase inhibitor p27 were observed in SNT16, KHYG1, KAI3, and SNK6 cells following treatment with tofacitinib for 24 h (Figure 4E). Furthermore, reduced levels of CDK2 or cyclin D3, which are associated with the G1 checkpoint, were observed in SNT15, KHYG1, and SNK6 (Figure 4E). These combined results suggest that tofacitinib induces G1 cell-cycle arrest in T and NK cell lines resulting in inhibition of proliferation.

### Tofacitinib suppresses the expression of LMP1 and EBNA1 in EBV-positive T and NK-cell lines

**Figure 5:**
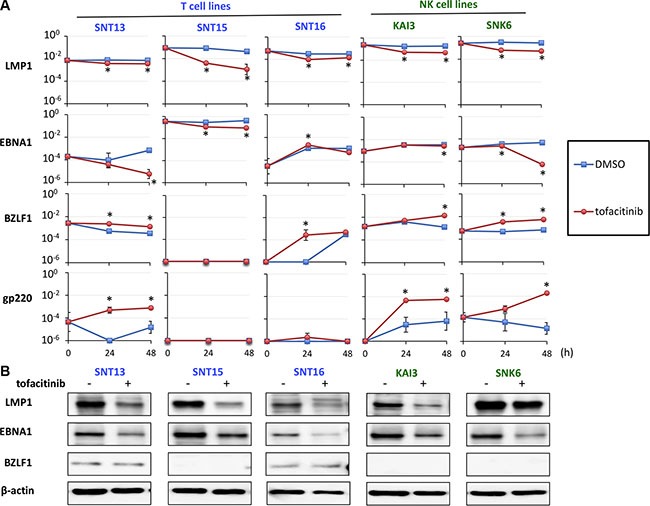
Effects of tofacitinib on the expression of EBV-encoded genes in EBV-positive T and NK cell lines (**A**) EBV-positive T cell lines (SNT13, SNT15, and SNT16) and EBV-positive NK cell lines (KAI3 and SNK6) were treated with 5 μM tofacitinib and harvested at 0, 24 and 48 h to evaluate gene expression using real-time RT-PCR. BZLF1 is an immediate early gene and gp350/220 is a late gene in the lytic infection cycle of EBV. LMP1 and EBNA1 are latent EBV genes. β2-Microglobulin was used as an internal control and as a reference gene for relative quantification and was assigned an arbitrary value of 1 (10°). Values are geometric means ± SE of the results from six replicate experiments. *P < 0.05 as compared with DMSO-treated cells. (**B**) SNT13, SNT15, SNT16, KAI3 and SNK6 cell lines were treated with 5 μM tofacitinib for 48 h, and cell lysates were immunoblotted for LMP1, EBNA1 and BZLF1. Actin was blotted as a loading control.

We next analyzed the effect of tofacitinib on the expression of EBV-encoded genes in T and NK cell lines. Expression of the following four viral genes was analyzed using real-time RT-PCR: lytic genes encoding BZLF1 and gp350/220 and latent genes encoding EBV nuclear antigen (EBNA) 1, and latent membrane protein (LMP) 1. The expression of LMP1 and EBNA1, which are known oncogenes or anti-apoptotic genes, was significantly decreased in SNT13, SNT15, KAI3, and SNK6 cell lines by treatment with tofacitinib (Figure 5A). In contrast, the expression of BZLF1 and gp350/220, which are an immediate-early gene and a late gene in the lytic infection cycle, respectively, was significantly increased in SNT13, KAI3, and SNK6 cell lines by treatment with tofacitinib (Figure 5A). Additionally, we evaluated the expression of LMP1, EBNA1, and BZLF1 proteins by immunoblotting. LMP1 and EBNA1 protein levels were decreased in all cell lines tested by treatment with tofacitinib (Figure 5B). In contrast to the results obtained using RT-PCR, BZLF1 protein levels were not increased in any cell line by treatment with tofacitinib (Figure 5B).

### Tofacitinib inhibits the growth of an established tumor in NOG mice

**Figure 6:**
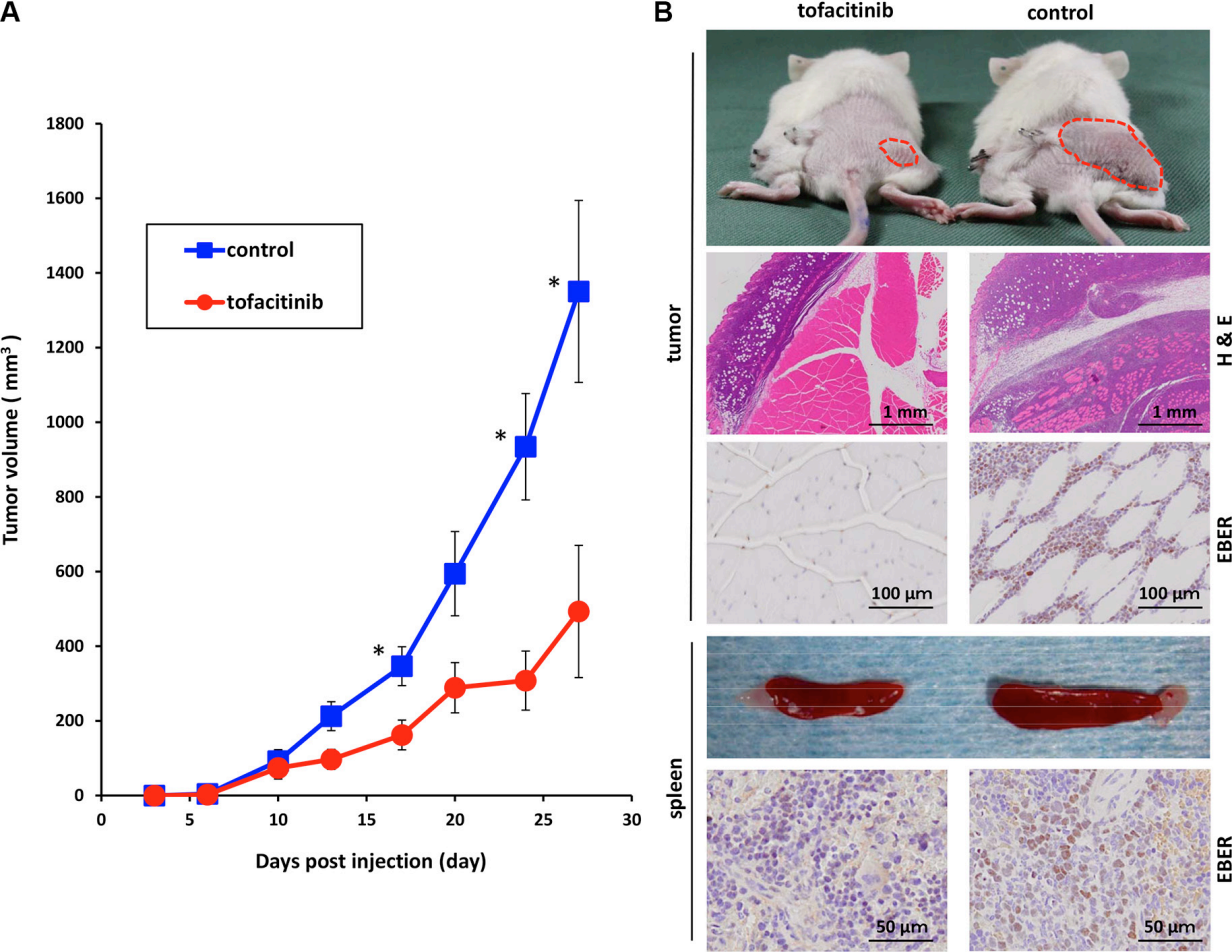
Effects of tofacitinib on tumor cell growth and proliferation in the murine xenograft model (**A**) Tofacitinib inhibited the growth of subcutaneous xenograft tumors in NOG mice. SNT15 cells (2 ×10^6^ cells per flank) were subcutaneously inoculated into the flanks of mice. On the day of tumor inoculation, treatment was started using subcutaneous mini-osmotic pumps and tofacitinib (30 mg/kg/day) was administered for 4 weeks. Tumor size was quantified twice a week. *P < 0.05; *n* = 6 mice for each group. (**B**) Representative images of tumor-bearing mice and removed spleens are shown after 4 weeks of treatment with tofacitinib or vehicle (control). The hematoxylin/eosin-stained section shows tumor infiltration into the subcutaneous lesion wall. EBER *in situ* hybridization shows infiltration into the subcutaneous lesion wall and spleen, respectively.

We further extended our studies to an *in vivo* xenograft model to validate the significance of the *in vitro* findings. We subcutaneously inoculated 2 × 10^6^ SNT15 cells into mice. On the day of tumor inoculation, mini-osmotic pumps were implanted and vehicle alone or tofacitinib (30 mg/kg/day) was delivered for 28 days. Subcutaneous inoculation of SNT15 cells into NOD/Shi-scid/IL-2Rγ^null^ (NOG) mice resulted in tumor formation at the site of injection in all mice except for one treated mouse. The mice generally tolerated tofacitinib with no apparent toxicity such as death, weight loss or not doing well throughout the experiment. An antitumor effect of tofacitinib on tumor growth was evident on day 13, and tumor growth was significantly suppressed in the tofacitinib-treated group versus the control group at the end of the experiment (*P* < 0.05; Figure 6A). The topmost images of Figure 6B are representative images of tofacitinib-treated and –untreated tumor-bearing mice. Hematoxylin/eosin staining and EBV-encoded small RNA (EBER) *in situ* hybridization showed tumor cell invasion into subcutaneous tissue in the untreated mice, whereas no invasion was observed in the tofacitinib-treated mice. EBER-positive cells, whose nuclei are stained brown, extensively infiltrate the muscle layer of the untreated mouse (Figure 6B). Furthermore, the untreated mice developed significant splenomegaly, and infiltration of many EBER-positive cells was observed in the spleen (Figure 6B).

### The JAK3/STAT5 pathway is activated in EBV-infected cells from a patient with EBV-associated T cell lymphoma

**Figure 7:**
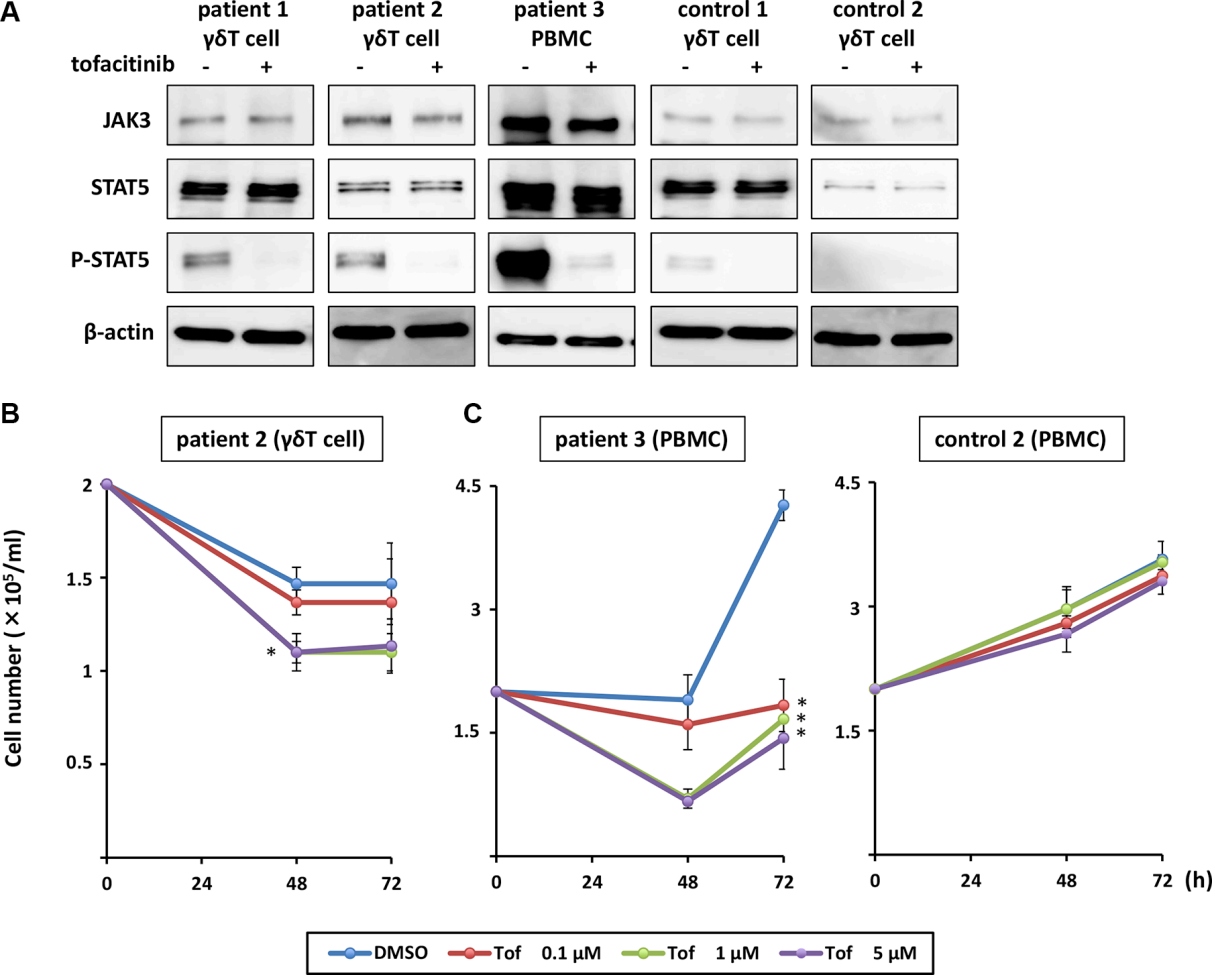
Effects of tofacitinib on JAK3/STAT5 pathway components and growth in EBV-infected cells isolated from patients with EBV-associated T cell lymphoma (**A**) γδ T cells and peripheral blood mononuclear cells (PBMCs) were treated with 1 μM tofacitinib for 24 h and cell lysates were immunoblotted for the indicated proteins. (**B**) γδ T cells isolated from patients with EBV-associated T cell lymphoma were treated with the indicated concentrations of tofacitinib, and viable cells were counted using the trypan blue exclusion test. *P < 0.05 as compared with DMSO-treated cells. (**C**) PBMCs isolated from a patient with EBV-associated T cell lymphoma or from a healthy donor (control) were treated with the indicated concentrations of tofacitinib, and viable cells were counted using the trypan blue exclusion test. Values are means ± SE of the results from triplicate experiments using patient samples. *P < 0.05 as compared with DMSO-treated cells.

Finally, we investigated the *ex vivo* effect of tofacitinib on lymphocytes from patients with hydroa vacciniforme-like lymphoma, which was recently defined as an EBV-associated T cell lymphoma. We separated γδ T cells, which harbor EBV, from the peripheral blood mononuclear cells (PBMCs) of two patients (Patient 1 and Patient 2) and a healthy donor (control) using magnetic sorting, and confirmed activation of the JAK3/STAT5 pathway in these cells. Phospho-STAT5 was detected in the patient samples and treatment of these cells with tofacitinib inhibited STAT5 phosphorylation (Figure 7A). Additionally, γδ T cells from Patient 2 were exposed to 0.1 to 5 μM of tofacitinib, and cell counts were determined after 48 and 72 h. Because the γδ T cells were too fragile to proliferate *ex vivo*, a significant growth-inhibitory effect of tofacitinib was not confirmed. However, the cell number of tofacitinib-treated cells decreased more rapidly than that of untreated cells (Figure 7B). We then cultured PBMCs from another patient with EBV-associated T cell lymphoma (Patient 3) and from a healthy donor with IL-2-supplemented media. Tofacitinib significantly inhibited the growth of the PBMCs from the patient compared to DMSO, while it only had a weak inhibitory effect on the growth of the PBMCs from the healthy donor (Figure 7C).

## DISCUSSION

In this study, we demonstrated that the JAK3/STAT5 pathway is activated in T/NK cell lines from EBV-associated lymphoma, and that the inhibition of such activation by tofacitinib results in a decrease in cell growth. Among the T cell lines tested, anti-tumor effects of tofacitinib were seen only for the EBV-positive cell lines, whereas both EBV-positive and -negative NK cell lines were sensitive to tofacitinib. Most of the cell lines whose proliferation was inhibited by tofacitinib were dependent on IL-2 for cell growth. Considering that IL-2 is a major cytokine for activation of the JAK3/STAT5 pathway, it was expected that IL-2 dependent cell lines would be sensitive to tofacitinib [20]. However, JAK3/STAT5 pathway activation and cell growth inhibition by tofacitinib were also observed in the IL-2 independent SNT16, EBV-positive T cell line. Furthermore, in a comparison of the EBV-positive NK cell line, TL1, with the EBV-negative parental NK cell line, NKL, the TL1 cells were more sensitive to tofacitinib than the NKL cells, suggesting that EBV infection could increase sensitivity to tofacitinib.

LMP1, which is a major oncoprotein of EBV, promotes activation of various signaling pathways, such as TRAF, NF-κB, and JNK signaling, leading to cell proliferation. On the other hand, EBNA1 plays an important role in the initiation of latent-cycle EBV DNA replication, transcriptional activation of other EBV latent proteins, and cellular transformation [21]. It has been reported that JAK3 can bind to LMP1 and is responsible for the activation of STAT proteins, although this finding is still controversial [22–27]. Interestingly, Chen *et al.* reported that activation of the JAK/STAT pathway promoted the expression of the LMP1 protein and vice versa, and proposed a positive feedback loop of LMP1 expression and STAT activation [25]. In the present study, we demonstrated that tofacitinib decreased the protein levels of LMP1 and EBNA1 whereas, decreases in the mRNA levels of these proteins were modest or not significant in some cell lines. Expressions of mRNAs and the corresponding proteins sometimes do not correlate well, although the reason is unclear. Tofacitinib may increase the degradation of EBV proteins possibly through enhancement of their ubiquitination [28].

In the xenograft model, we demonstrated that subcutaneous inoculation of SNT15 cells (EBV-positive T cells) into NOG mice resulted in tumor formation, and that tofacitinib significantly suppressed the growth and invasion of the tumor without remarkable side effects. Furthermore, we found that EBV-infected γδ T cells of patients with EBV-associated T cell lymphoma showed activation of the JAK3/STAT5 pathway. Patients with EBV-associated T cell lymphoma are occasionally treated with stem cell transplantation because most EBV-associated T cell lymphoma cases are refractory and resistant to conventional chemotherapies. Our results indicate that inhibition of the JAK3/STAT5 pathway might be a promising strategy for treatment of EBV-associated T cell lymphoma.

Previous studies have reported that inhibition of the JAK3/STAT5 pathway induced apoptosis of tumor cells; however, its effect on the cell cycle has not been fully investigated [14, 15]. In this study, we found that tofacitinib induced G1 cell cycle arrest and inhibited cell growth in all of the EBV-positive T and NK cell lines tested. On the other hand, tofacitinib induced modest or no apoptosis in the EBV-positive cell lines tested. Although tofacitinib significantly increased the number of apoptotic cells in SNT15 compared to DMSO treatment, the frequency of apoptotic SNT15 cells was much smaller than that of apoptotic KHYG cells. Furthermore, tumor growth was not completely inhibited by tofacitinib in the murine xenograft model of SNT15, suggesting that the effect of single-agent therapy could be limited.

In conclusion, we demonstrated that tofacitinib has the potential to be a new therapeutic agent against EBV-associated T and NK lymphoma. Although there is still concern regarding side effects of tofacitinib treatment, very strong adverse side effects have not been reported and clinical trials of tofacitinib for the treatment of many diseases are currently being conducted [29, 30]. Further future research regarding the use of this JAK3 inhibitor as an anticancer agent is warranted.

## MATERIALS AND METHODS

### Cell lines and reagents

The cell lines used were as follows: LCL cells were established by infection of B cell with EBV, strain B95-8. SNT13, SNT15 and SNT16 are EBV-positive T cell lines, SNK6 and KAI3 are EBV-positive NK cell lines, Jurkat and MOLT4 are EBV-negative T cell lines, and BJAB and KHYG1 are EBV-negative B- and NK-cell lines, respectively. SNT13, SNT15, SNT16, SNK6 and KAI3 cells were derived from patients with CAEBV or EBV-associated NK/T cell lymphoma [31–33]. The NKL cell line was derived from a patient with NK cell leukemia, and the TL1 cell line was established from NKL cells infected with an Akata-transfected recombinant EBV strain carrying a neomycin resistance gene [34]. Jurkat, MOLT4, LCL, and BJAB cells were grown in RPMI-1640 supplemented with 10% heat-inactivated FBS, penicillin and streptomycin (complete medium). Complete medium supplemented with 100 U/ml human IL-2 was used for SNT13, SNT16, SNK6, KAI3, KHYG1, NKL and TL1. SNT15 cells were grown in serum-free medium supplemented with 250 IU/mL IL-2 (Artemis-2; Nihon Techno Service). IL-2 independent SNT16 cells were grown in the same medium without IL-2. The characteristics of each cell line are summarized in Table 1. Tofacitinib was purchased from Sigma-Aldrich and was dissolved in DMSO.

### Immunoblotting

Cells were lysed directly in sample buffer. Equal amounts of protein were subjected to SDS-PAGE, transferred to polyvinylidene difluoride (PVDF) membranes, and incubated with antibody. Antibodies against JAK3, STAT5, phospho-STAT5 (Tyr694), p27Kip1 and caspase-3 (Cell Signaling Technology); β-actin and PARP (Sigma-Aldrich); CDK2, cyclin D3 (BD Biosciences); LMP1 and EBNA1 (provided by Teru Kanda, Aichi Cancer Center, Aichi, Japan); and BZLF1 [35] were used for immunoblots.

### Cell numbers and viability

Cells were seeded in 24-well plates at a density of 2 × 10^5^/ml, and cell numbers were assayed by trypan blue exclusion using a Countess automated cell counter (Invitrogen). Experiments were performed at least in triplicate.

### Annexin V analysis of apoptosis

Apoptosis was measured using an Annexin V-PE/7-AAD apoptosis assay kit (BD Pharmingen Biosciences) in accordance with the manufacturer's instructions. Cells were analyzed using flow cytometry. Viable cells were defined as negative for Annexin V-phycoerythin (PE) and 7-aminoctinomycin (7-AAD) staining, whereas apoptotic cells were defied as positive for Annexin V-PE and negative for 7-AAD staining.

### Cell-cycle assay

Cells were treated with 5 μM of tofacitinib for 24 h, fixed with 70% ethanol, and then washed with ice-cold PBS. Fixed cells were treated with DNase-free RNase and stained with propidium iodide (Sigma-Aldrich). The stained cells were analyzed using flow cytometry and ModFit LT software (Verity Software House). Experiments were performed in triplicate.

### Real-time RT-PCR

Viral mRNA expression was quantified using RT−PCR, as described previously, using β2-microglobulin as an endogenous control and a reference gene for relative quantification [36, 37]. Each experiment was performed in triplicate.

### Xenograft model

NOG mice were obtained from the Central Institute for Experimental Animals (Kawasaki, Japan) and were maintained under specific pathogen-free conditions in the animal facility of Nagoya University (Nagoya, Japan). SNT15 cells (2 × 10^6^ cells per flank) were suspended in a volume of 100 μL and subcutaneously inoculated into the right flanks of mice. Mini-osmotic pumps (ALZET) were implanted into the left flanks on the same day. Tofacitinib was dissolved in a sterile solution of 50% DMSO, 10% PEG and 40% saline solution and was delivered at a flow rate of 0.5 μL/h for four weeks [38–40]. Mice were randomized to drug –related or control groups. Tumor size was quantified with calipers twice a week, and peripheral blood was obtained every week. All animal experiments were approved by the University Committee in accordance with the Guidelines for Animal Experimentation at Nagoya University.

### Immunohistochemistry

All mice were humanely killed and the body tissues including tumors, brain, heart, kidney, lung and spleen were removed. Formaldehyde-fixed, paraffin-embedded tissues were cut into serial sections about 3-μm thick. Sections were then stained with hematoxylin and eosin and were stained for EBER by *in situ* hybridization using the EBER PNA Probe/FITC (Y5200; Dako) and the VECTASTAIN Elite ABC kit (Vector Laboratories), according to the manufacturers' protocols.

### Human samples and magnetic sorting

PBMCs were obtained from 3 patients with hydroa vacciniforme-like lymphoma and from 2 healthy donors. Hydroa vacciniforme-like lymphoma was diagnosed according to the World Health Organization (WHO) criteria [41]. EBV-infected cells were determined as γδ T cells in these patients by using a flow cytometric *in situ* hybridization assay [42]. γδ T cell fractions were separated by magnetic sorting using the TCRγ/δ^+^ T Cell Isolation kit (Miltenyi Biotec). Informed consent was obtained from all participants or their guardians according to the Declaration of Helsinki. This study was approved by the Institutional Review Board of Nagoya University Hospital.

### Statistical analysis

Statistical analyses of cell proliferation, cell cycle, and tumor volume were performed using the Mann-Whitney *U* test. Probability values of < 0.05 were considered to be statistically significant.
